# Efficient isolation method for high‐quality genomic DNA from cicada exuviae

**DOI:** 10.1002/ece3.3398

**Published:** 2017-09-05

**Authors:** Hoa Quynh Nguyen, Ye Inn Kim, Amaël Borzée, Yikweon Jang

**Affiliations:** ^1^ Department of Life Science Ewha Womans University Seoul Korea; ^2^ Department of Biological Science Seoul National University Seoul Korea

**Keywords:** cicada exoskeleton, *Crytptotympana atrata*, exuviae, noninvasive DNA sampling

## Abstract

In recent years, animal ethics issues have led researchers to explore nondestructive methods to access materials for genetic studies. Cicada exuviae are among those materials because they are cast skins that individuals left after molt and are easily collected. In this study, we aim to identify the most efficient extraction method to obtain high quantity and quality of DNA from cicada exuviae. We compared relative DNA yield and purity of six extraction protocols, including both manual protocols and available commercial kits, extracting from four different exoskeleton parts. Furthermore, amplification and sequencing of genomic DNA were evaluated in terms of availability of sequencing sequence at the expected genomic size. Both the choice of protocol and exuvia part significantly affected DNA yield and purity. Only samples that were extracted using the PowerSoil DNA Isolation kit generated gel bands of expected size as well as successful sequencing results. The failed attempts to extract DNA using other protocols could be partially explained by a low DNA yield from cicada exuviae and partly by contamination with humic acids that exist in the soil where cicada nymphs reside before emergence, as shown by spectroscopic measurements. Genomic DNA extracted from cicada exuviae could provide valuable information for species identification, allowing the investigation of genetic diversity across consecutive broods, or spatiotemporal variation among various populations. Consequently, we hope to provide a simple method to acquire pure genomic DNA applicable for multiple research purposes.

## 1 INTRODUCTION

Nondestructive sampling methods for DNA resources have recently attracted more attention from ethological, conservational, and population genetic studies. DNA extraction from specimens usually required scarifying essential sections of the insects such as leg, thorax, or head capsule. Such sampling methods could cause severe impacts on the species at both individual and population levels. Invasive sampling could have negative consequences on subsequent behavior and survival of sampled individuals. Extensive sampling is problematic for small colonies of social insects (Starks & Peters, 2002). Moreover, lethal sampling potentially decreases population size and alters population structure (Starks & Peters, 2002), which is harmful for the conservation of endangered species. Consequently, nondestructive sampling methods are in need for various genetic analyses (Châline, Ratnieks, Raine, Badcock, & Burke, 2004; Su et al., 2007).

Exuviae have been demonstrated to be reliable genetic sources for a variety of species, including popular taxa such as honey bees (Gregory & Rinderer, 2004), mosquitoes (Dhananjeyan et al., 2010), and scarabs (Lefort, Boyer, Worner, & Armstrong, 2012), and endangered species such as dragonflies (Keller, Brodbeck, & Holderegger, 2009; Monroe, Lynch, Soluk, & Britten, 2010) and tarantulas (Petersen et al., 2007). Cicada exuviae are exoskeletons that remain after molting of final instar nymphs. Such material can persist despite exposure to variable environmental conditions. One exuvia equals one successfully emerged adult individual. Exuviae can therefore serve as a useful source for both ecological and genetic studies. While cicada exuviae have been employed in various ecological studies, such as species identification (Lee, Oh, & Jang, 2012; Wei, Hou, & Li, 2014), estimation of population densities (Patterson, Massei, & Genov, 1997; Lee, Lin, & Wu, 2010; Kim, Oh, Chang, & Jang, 2014), species distribution (Rodenhouse, Bohlen, & Barrett, 1997), and estimation of emergence period (Sato & Sato, 2015), only a few studies have mentioned the employment of cicada exoskeleton as source of their genetic materials (Bouwer, Midgley, Timm, & Villet, 2014; de Oliveira, Felipe, Wallau, & Silva Loreto, 2009).

One of the main reasons for the rare application of cicada exuviae in molecular works is that the exoskeleton itself does not contain any genomic material. The cuticle plays the role of the insect exoskeleton, which is chemically composed of chitin, a polysaccharide polymer of *N*‐acetyl‐glucosamine, cuticular proteins, cuticular lipids, phenols, and quinones (Nation, 2008). Trace genomic DNA can be extracted from muscle tissues or metabolic waste products that the individual left on the inner side of the exoskeleton after molt (Nation, 2008). Another reason for the rare application of cicada exuviae is the presence of potential polymerase chain reaction (PCR) inhibiting substances in soil, such as humic acids. Genomic DNA extracted from cicada exuviae can therefore contain contaminants that inhibit the usage of those DNA samples in downstream applications such as amplification of target sequence (Baar et al., 2011; Braid, Daniels, & Kitts, 2003; Kermekchiev, Kirilova, Vail, & Barnes, 2009; Schrader, Schielke, Ellerbroek, & Johne, 2012; Straub, Pepper, & Gerba, 1995).

Our goals in this study are to evaluate protocols for DNA extraction from cicada exuviae regarding their quality and quantity of DNA yield and to suggest the best protocol for downstream applications. Six extraction protocols including available commercial kits and manual protocols were tested. We further identified those parts of the cicada exoskeleton from which high DNA yield was obtained.

## 2 METHODOLOGY

### 2.1 Sample collection

**Figure 1:**
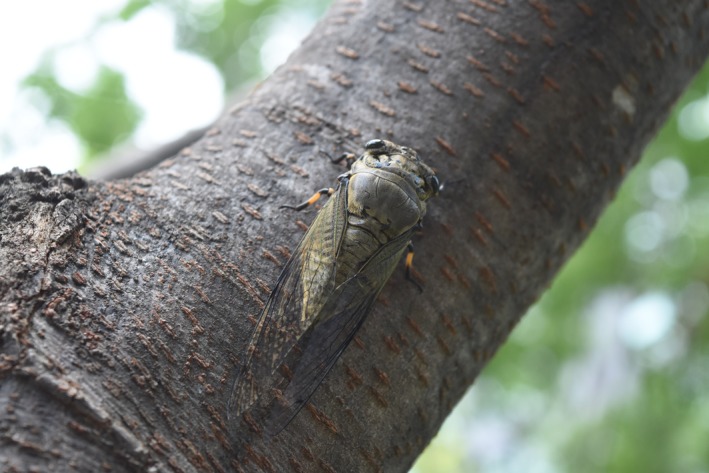
The black cicada (*Cryptotympana atrata*). This species is very common in urban areas in Korea. Photograph credit Yoonhyuk Bae

Exuviae of the black cicada (*Cryptotympana atrata*, Fig. 1) were collected in Gwangjin‐gu, Seoul, Korea (37.533415°N, 127.070493°E), on 11 July 2015. The sampling location was an apartment complex where multiple cicada species coexisted, that is, *C. atrata*,* Hyalessa fuscata*, and *Meimuna opalifera*. After field collection, samples were identified for species based on morphological characters (Lee et al., 2012) and were stored at ambient temperature. DNA extraction work on those exuviae was performed approximately 14 months after field collection.

### 2.2 DNA extraction procedure

Six manual and kit protocols were employed to extract DNA from cicada exuviae. Manual protocols included (1) ethanol precipitation using sodium chloride (EtSC), (2) ethanol precipitation using ammonium acetate (EtAA), and (3) chelex 5% (Ch5%). Kit protocols consisted of (4) LaboPass^™^ DNA Purification Kit (LP) (COSMO Genetech Co., Ltd., Seoul, South Korea), (5) DNeasy^®^ Blood and Tissue Kit (DN) (QIAGEN Group, Hilden, Germany), and (6) PowerSoil DNA Isolation Kit (PS) (MO BIO Laboratories, Inc., CA, USA). For each protocol, we randomly chose ten exuviae, regardless of sex, and divided each exuvia into four samples, each including a different exoskeleton part, that is, head, legs, thorax, and abdomen. Samples were separately inserted into 1.5‐ml microcentrifuge tubes. In total, 40 samples were used for each protocol.

All samples were homogenized using a pestle. To standardize among protocols, we incubated all samples in a thermo‐shaker at 2.5 xg for 20 hr. Cell lysis buffer and procedure of each protocol are shown in Table 1. For PS samples, additional 10‐min vortex mixing at maximum speed using a MO BIO Vortex Adapter was performed after incubation. The remaining exoskeletons were removed from each tube after incubation, and the tubes were centrifuged at 18,000 xg for 2 min. Each supernatant was carefully transferred to a new 1.5‐ml tube, avoiding the transfer of the pellet. Subsequent steps were performed following manufactures' protocols for kits. For EtSC samples, precipitation of cell debris was performed by adding 166.7 μl of 6 mol/L NaCl to each tube followed by centrifuging at maximum speed for 10 min, after which the top supernatant layer was transferred to a new 1.5‐ml tube with 1 ml of cold 100% ethanol and incubated overnight at −20°C. The samples were washed twice by adding 800 μl 70% ethanol, via briefly vortexing the sample followed by carefully pipetting off the supernatant without dislodging the DNA pellet. Pellets were left to dry in air for approximately 30 min and then resuspended in ultrapure water (Biosesang Inc., Gyeonggi‐do, Republic of Korea). For EtAA samples, a precipitation step was carried on via addition of 200 μl of 4 mol/L ammonium acetate followed by centrifugation at 18,000 xg for 20 min before transferring the top supernatant layer into a new 1.5‐ml tube. The samples were washed with ethanol as described for EtSC samples and resuspended in ultrapure water (Biosesang Inc., Gyeonggi‐do, Republic of Korea). Following the Ch5% protocol, the samples after incubation were further incubated at 100°C for 15 min and then centrifuged at 14,000 xg for 4 min, and the top layer supernatant was transferred to a new 1.5‐ml tube.

**Table 1:** Cell lysis buffers and DNA extraction procedures of six protocols: ethanol precipitation using sodium chloride (EtSC), ethanol precipitation using ammonium acetate (EtAA), Chelex 5% (Ch5%), LaboPass^™^ Genomic DNA Purification Kit (LP), DNeasy^®^ Blood and Tissue Kit (DN), and PowerSoil DNA Isolation Kit (PS). For each protocol, 40 samples from 10 cicada exuviae were employed

Protocol	Volume of cell lysis buffer	Volume of Proteinase K (20 mg/ml)	Incubation temperature (^o^C)
Ethanol precipitation using sodium chloride	600 μl of TNES buffer (Tris pH 7.5 10 mmol/L, NaCl 400 mmol/L, EDTA 100 mmol/L, and SDS 0.6%)	70 μl	50
Ethanol precipitation using ammonium acetate	600 μl of digestion buffer (NaCl 50 mmol/L, Tris pH 8.0 50 mmol/L, EDTA pH 8.0 20 mmol/L, and SDS 1%)	6 μl	55
Chelex 5%	360 μl of chelex 5% buffer	80 μl	57
LaboPass^™^ Genomic DNA Purification Kit	800 μl buffer TL	40 μl	56
DNeasy^®^ Blood and Tissue Kit	360 μl buffer ATL	40 μl	56
PowerSoil DNA Isolation Kit	60 μl Solution C1	60 μl	65

### 2.3 Acquisition of UV–Vis spectra of DNA concentration

Extracted DNA samples were examined by gel electrophoresis in 1% agarose gel (Biopure, Genomic Base, Seoul, Republic of Korea) visualized on an UltraSlim LED Illuminator (MaestroGen Inc., Hsinchu, Taiwan) using MaestroSafe Nucleic Acid loading dye (MaestroGen Inc., Hsinchu, Taiwan). Quantity and quality of DNA samples were measured using a NanoDrop^™^ 2000/2000c spectrophotometer (Thermo Fisher Scientific Inc., Delaware, USA). In particular, the ratio of 260/280 indicated the presence of organic contaminants such as protein or phenol that strongly absorb at 280‐nm wavelength; likewise, 260/230 indicated the appearance of other contaminants absorbing at 230‐nm wavelength such as EDTA or carbohydrates. A DNA sample was considered pure when both 260/280 and 260/230 ratios ranged between 1.8 and 2.0. For Ch5% samples, due to lack of baseline buffer, we used original Chelex 5% as baseline buffer, and the measurement of two ratios was employed only for purity comparison among protocols, but was not included in the statistical analysis.

### 2.4 PCR amplification and purification

**Figure 2:**
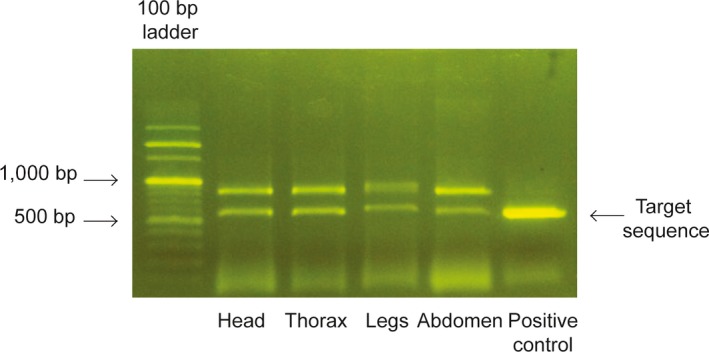
Gel electrophoresis of 16S gene amplified in four genomic DNA samples extracted by PowerSoil DNA Isolation Kit and one positive control of genomic DNA extracted from tissue of the same species. Three microliters of each PCR product were loaded on 1.5% agarose gel. Successful PCR amplification should exhibit bands of a target size similar to the positive control

Five hundred bp of the 16S region was amplified using two primers: LR‐J‐12887 (5′‐CCGGTCTGAACTCAGATCACGT‐3′) and LR‐N‐13398 (5′‐CGCCTGTTTAACAAAAACAT‐3′) (Simon et al., 1994) by Takara Ex Taq (Takara Korea Biomedical Inc., Seoul, Republic of Korea). For each PCR, 40 samples of each extraction protocol were run along with one negative control of ultrapure water (Biosesang Inc., Gyeonggi‐do, Republic of Korea) and one positive control of genomic DNA extracted from tissue of *C. atrata*. A total of 25 μl amplified sample consisted of 2 μl template DNA and 23 μl of master mix (0.125 μl of Takara Ex Taq^™^ 5 U/μl, 2.5 μl of 10× Buffer, 2 μl of dNTP Mix 2.5 mmol/L, 2 μl of MgCl_2_ 25 mmol/L, 2 μl of each primer 10 mmol/L and ultrapure water (Biosesang Inc., Gyeonggi‐do, Republic of Korea). PCR amplification initiated by 1‐min initial denaturation at 94°C, followed by 30 cycles of 30 s denaturation at 94°C, 1‐min annealing at 56°C, and 1‐min elongation at 72°C, finally completed by 2‐min terminal elongation at 72°C. Three microliters of each PCR product were loaded on 1.5% agarose gel and visualized using the same loading dye and LED illuminator as described above. Samples with bands that appeared at 600‐bp size, as in the positive control band (Fig. 2), were considered as PCR success and were used for the gel purification procedure. We labeled 1 for successful amplification and 0 for amplification failure. Gel bands were excised using a sterile scalpel, and gel purification was conducted using a QIAquick^®^ Gel Extraction Kit (QIAGEN Group, Hilden, Germany). All samples were sequenced both in forward and in reverse directions by COSMO Genetech Company (COSMO Genetech Co., Ltd., Seoul, South Korea), and sequencing success was labeled 1 as successful sequencing and 0 for sequencing failure.

### 2.5 Statistical analysis

Generalized linear model (GLM) was performed to test for effects of extraction protocols and exoskeleton parts on DNA quantity and purity ratios. Dependent variables included DNA concentration (ng/μl), A260/280 ratios, and A260/230 ratios. Fixed factors included five extraction protocols and four exoskeleton parts. Ch5% samples were excluded from statistical analysis due to lack of buffer for DNA concentration measurements and due to lack of gel band of expected size. Normal distribution with identity link function was applied on A260/280 ratio, whereas Gamma distribution using log link function was applied on DNA concentration and A260/230 ratio. We removed negative A260/230 ratio values and an extreme outlier as they represented inaccurate measurements. The assumption on homoscedasticity was examined by visualizing the plot of predicted value against standardized residuals. Multiple pairwise comparisons were carried out using the Sidak test to identify significant differences across protocols and also among parts.

As only samples extracted from PowerSoil Kit provided PCR bands of expected size and target sequences, binomial logistic regression was performed to test for factors affecting amplification and sequencing success. Dependent variable was either amplification or sequence success. Part was employed as independent variable, whereas DNA concentration, A260/280 and A260/230 as covariates. All statistical analyses were conducted using SPSS 22 (IBM Corp; New York, USA).

## 3 RESULTS

### 3.1 UV–Vis spectra of DNA samples

**Figure 3:**
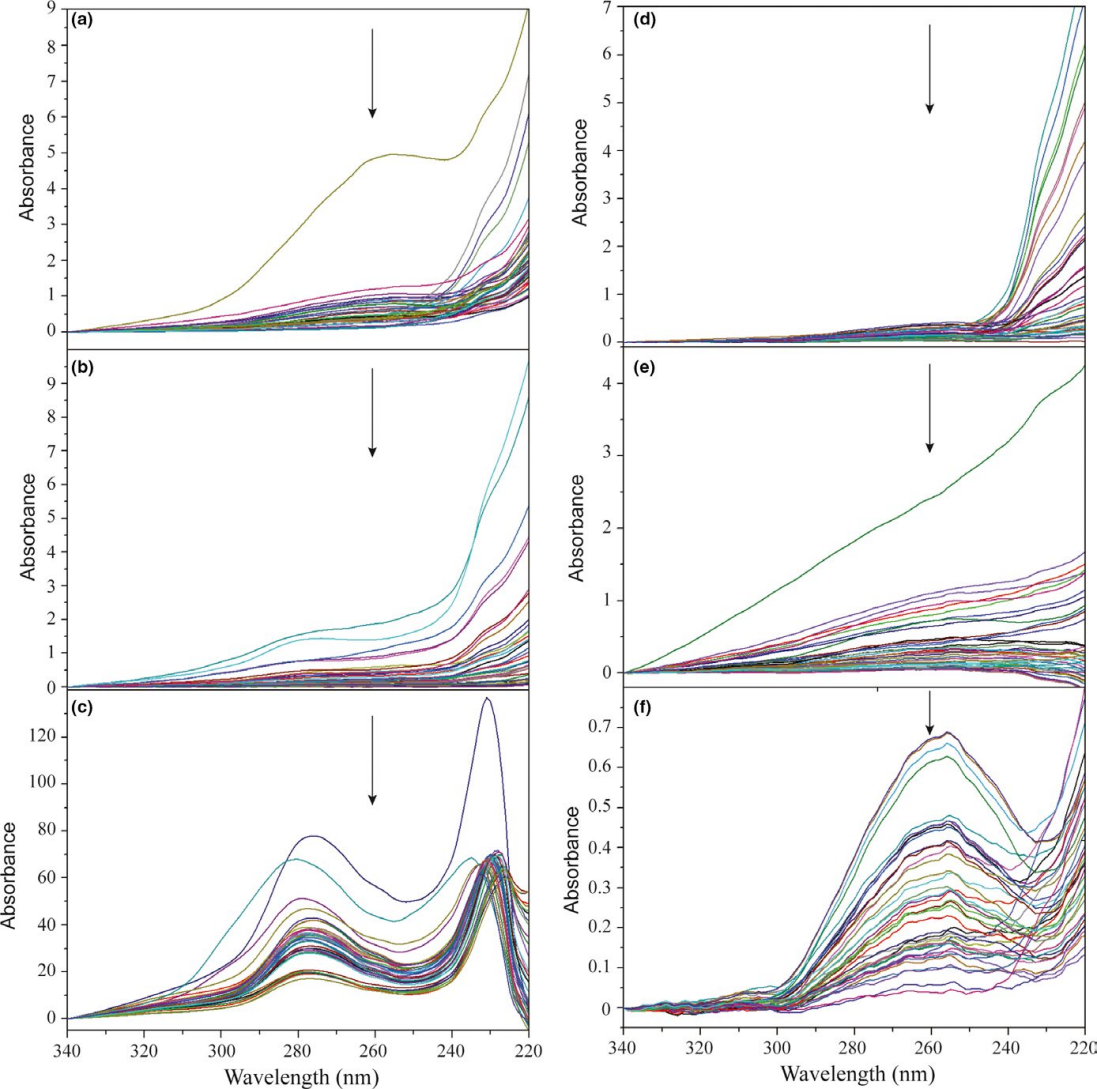
UV–Vis spectra of DNA samples extracted by six protocols: (a) Ethanol precipitation using sodium chloride, (b) ethanol precipitation using ammonium acetate, (c) Chelex 5%, (d) LaboPass^™^ DNA Purification Kit, (e) DNeasy^®^ Blood and Tissue Kit, and (f) PowerSoil DNA Isolation Kit. Peaks at 260 nm (demonstrated by arrows) indicate the absorbance wavelength of genomic DNA, while the lack of peaks at this wavelength implies organic contaminants such as humic acids. In six protocols, only samples extracted by PowerSoil kit show clear peaks at 260 nm

UV–Vis spectra of 40 DNA samples extracted by six protocols are shown in Fig. 3. Among those protocols, only samples extracted by PowerSoil DNA Isolation kit (Fig. 3f) show clear peaks at 260 nm, which corresponds to the absorbance wavelength of DNA, as well as humic acids available in soil.

### 3.2 DNA concentration

**Figure 4:**
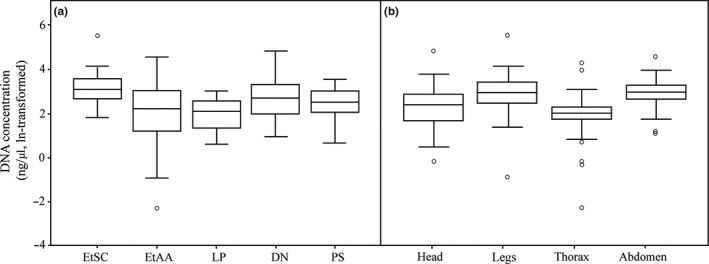
Comparison of the DNA concentration (ng/ul, ln‐transformed). (a) DNA samples extracted from five DNA extraction protocols: Ethanol precipitation using sodium chloride (EtSC), ethanol precipitation using ammonium acetate (EtAA), LaboPass^™^ DNA Purification Kit (LP), DNeasy^®^ Blood and Tissue Kit (DN), and PowerSoil DNA Isolation Kit (PS). (b) DNA samples extracted from four exoskeleton parts: head, legs, thorax, and abdomen. For each box plot, the line within the box represents the mean; the top and bottom lines represent 75 and 25 percentiles of the data, respectively; top and bottom whiskers represent 95 and 5 percentiles, respectively; circles represent outliers

Results of GLM showed that both protocol and part contributed as significant factors affecting DNA concentration (for protocol Wald χ^2^ = 47.62, *df* = 4, *p *<* *.001, for part Wald χ^2^ = 37.28, *df* = 3, *p *<* *.001). A comparison across protocols (Table 2, Fig. 4a) showed that DNA concentrations of EtSC samples were significantly higher than those of other protocols (*p *<* *.05), except DN (*p *=* *.963). The DNA concentration of EtAA samples was significantly lower than that of EtSC samples (*p *=* *.011), but comparable to other protocols (*p *>* *.05). The DNA concentration of DN samples was also significantly higher than that of LP samples (*p *<* *.001). No difference in DNA concentration was found between LP and PS samples (*p *>* *.05). Pairwise comparisons of exoskeleton parts (Table 3, Fig. 4b) showed significantly higher DNA concentrations in legs and abdomen compared to head (*p *<* *.05) and thorax (*p *<* *.05). No difference in DNA concentration was found between legs and abdomen (*p *>* *.05) or between head and thorax (*p *>* *.05).

**Table 2:** Multiple Sidak pairwise comparisons of DNA concentration, A260/280 and A260/230 ratios across five protocols. Samples extracted by Chelex 5% were excluded due to lack of baseline buffer. The protocol was determined as a significant factor in the variation in DNA concentration, A260/280 and A260/230 ratios using generalized linear models. For each protocol, 10 exuviae, each divided into four exoskeleton parts, were randomly chosen. Significant *p* values are shown in bold. I–J: difference between protocol I and protocol J, *SE*: standard error

Protocol I	Protocol J	ln(DNA concentration)			A260/280 ratio			ln(A260/230 ratio)		
		I–J	*SE*	*p*	I–J	*SE*	*p*	I–J	*SE*	*p*
Sodium chloride	Ammonium acetate	12.13	3.72	**.011**	0.25	0.04	**<.001**	0.02	0.06	1
	Labopass	17.61	3.44	**<.001**	−0.02	0.04	1	−0.10	0.07	.828
	DNeasy	4.71	4.36	.963	0.16	0.05	**.005**	−0.89	0.16	**<.001**
	PowerSoil	13.30	3.64	**.003**	−0.30	0.04	**<.001**	−0.76	0.13	**<.001**
Ammonium acetate	Labopass	5.48	2.1	.087	−0.27	0.04	**<.001**	−0.12	0.07	.623
	DNeasy	−7.42	3.46	.277	−0.09	0.04	.379	−0.91	0.15	**<.001**
	PowerSoil	1.17	2.41	1	−0.55	0.04	**<.001**	−0.77	0.13	**<.001**
Labopass	DNeasy	−12.90	3.09	**<.001**	0.18	0.04	**.001**	−0.79	0.16	**<.001**
	PowerSoil	−4.31	1.95	.243	−0.28	0.04	**<.001**	−0.65	0.14	**<.001**
DNeasy	PowerSoil	8.59	3.31	.091	−0.46	0.04	**<.001**	0.14	0.19	.999

**Table 3:** Multiple Sidak pairwise comparisons of DNA concentration, A260/280 and A260/230 ratios across four exoskeleton parts. Samples extracted by Chelex 5% were excluded due to lack of baseline buffer. The exoskeleton part was determined to be a significant factor to the variation in DNA concentration, A260/280 and A260/230 ratios using generalized linear models. Each part was extracted from 10 random exuviae by five different protocols. Significant *p* values are shown in bold. I–J: difference between protocol I and protocol J, *SE*: standard error

Part I	Part J	ln(DNA concentration)			A260/280 ratio			ln(A260/230 ratio)		
		I–J	*SE*	*p*	I–J	*SE*	*p*	I–J	*SE*	*p*
Head	Legs	−**8.64**	**2.90**	**.017**	0.08	0.04	.177	−0.04	0.10	.999
	Thorax	3.64	1.89	.286	0.10	0.04	.058	0.21	0.09	.102
	Abdomen	−**8.17**	**2.87**	**.026**	**0.11**	**0.04**	**.035**	0.06	0.10	.992
Legs	Thorax	**12.28**	**2.69**	**<.001**	0.02	0.04	.998	**0.25**	**0.09**	**.039**
	Abdomen	0.47	3.42	1	0.02	0.04	.991	0.10	0.10	.903
Thorax	Abdomen	−**11.81**	**2.62**	**<.001**	0.01	0.04	1	−0.15	0.08	.374

### 3.3 A260/280 ratio

**Figure 5:**
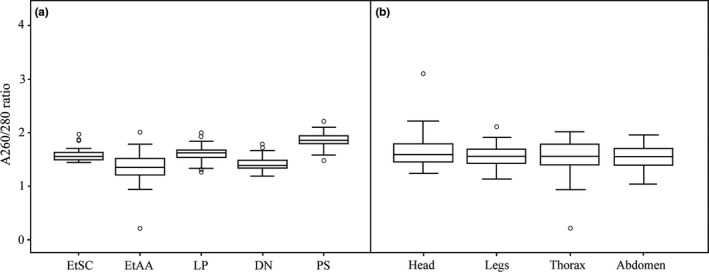
Comparison of the A260/280 ratios. (a) DNA samples extracted from five DNA extraction protocols: ethanol precipitation using sodium chloride (EtSC), ethanol precipitation using ammonium acetate (EtAA), LaboPass^™^ DNA Purification Kit (LP), DNeasy^®^ Blood and Tissue Kit (DN), and PowerSoil DNA Isolation Kit (PS). (b) DNA samples extracted from four exoskeleton parts: head, legs, thorax, and abdomen. For each box plot, the line within the box represents the mean; the top and bottom lines represent 75 and 25 percentiles of the data, respectively; top and bottom whiskers represent 95 and 5 percentiles, respectively; circles represent outliers

Similar to DNA concentration, both protocol and exoskeleton part were significant factors on A260/280 ratio (for protocol Wald χ^2^ = 182.35, *df* = 4, *p *<* *.001, for part Wald χ^2^ = 9.72, *df* = 3, *p *=* *.021). Only samples extracted using the PS protocol possessed an A260/280 ratio within 1.8–2.0 purity range (1.89 ± 0.03, estimated mean ± *SE*), and they were also significantly higher than other samples in this ratio (*p *<* *.001) (Table 2, Fig. 5a). Ratios of LP (1.60 ± 0.03) and EtSC samples (1.58 ± 0.03) were similar (*p *=* *1), and both of them were significantly higher than ratios of DN (1.44 ± 0.03) (*p *<* *.01) and EtAA samples (1.34 ± 0.03) (*p *<* *.001). In terms of exoskeleton parts, head, legs, and thorax were analogous with respect to A260/280 ratio (*p *>* *.05) (Table 3, Fig. 5b); the ratio of thorax was significantly higher than that of abdomen (*p *=* *.037).

### 3.4 A260/230 ratio

**Figure 6:**
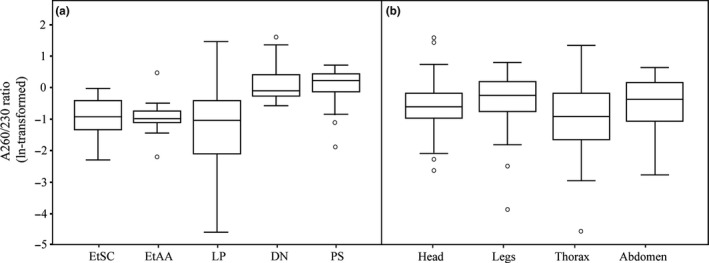
Comparison of the A260/230 ratios (ln‐transformed). (a) DNA samples extracted from five DNA extraction protocols: ethanol precipitation using sodium chloride (EtSC), ethanol precipitation using ammonium acetate (EtAA), LaboPass^™^ DNA Purification Kit (LP), DNeasy^®^ Blood and Tissue Kit (DN) and PowerSoil DNA Isolation Kit (PS). (b) DNA samples extracted from four exoskeleton parts: head, legs, thorax, and abdomen. For each box plot, the line within the box represents the mean; the top and bottom lines represent 75 and 25 percentiles of the data, respectively; top and bottom whiskers represent 95 and 5 percentiles, respectively; circles represent outliers

Analogous significant effects of protocol and exoskeleton part on A260/230 ratio were determined (for protocol Wald χ^2^ = 110.37, *df* = 4, *p *<* *.001, for part Wald χ^2^ = 9.13, *df* = 3, *p *=* *.028). DN and PS samples were found to be the highest in this ratio (the former 1.32 ± 0.15, the latter 1.18 ± 0.12), and their ratios were significantly higher than ratios of other samples (*p *<* *.001) (Table 2, Fig. 6a). Samples extracted by other protocols were similar in A260/230 ratio (LP samples 0.53 ± 0.06, EtSC samples 0.42 ± 0.05, and EtAA samples 0.41 ± 0.04) (*p *>* *.05 in multiple pairwise comparisons). With respect to exoskeleton parts, head, legs, and abdomen showed similar ratios (*p *>* *.05), although the ratio of thorax was lower than that of Legs (*p *=* *.039) (Table 3, Fig. 6b).

### 3.5 Amplification/sequencing success

We compared all sequences to the sequence GU344091, which is a partial sequence of 16S large subunit ribosomal RNA gene of *C. atrata* voucher MHV1476, and the pairwise identity ranges from 97.2% to 99.3%. PowerSoil was the only protocol adequate for generating PCR bands of the expected size (34 bands in a total of 40 samples) as well as providing sequencing success (12 successful sequences in a total of 40 sequencings). Nevertheless, binomial logistic regression models were not found to be significant (for amplification success, likelihood ratio χ^2^ = 10.05, *df* = 6, *p *=* *.123; for sequencing success likelihood ratio χ^2^ = 3.27, *df* = 6, *p *=* *.775). None of the factors were significant to either amplification success or sequencing attempts (*p *>* *.05). In total of 10, the samples that showed amplified targeted bands included nine head samples, nine thorax samples, eight leg samples, and eight abdomen samples. This indicates that most of the samples extracted by PowerSoil kit can generate the expected sequence.

## 4 DISCUSSION

In this study, we compared six methods for the DNA extraction from cicada exuviae. Among those, PowerSoil DNA Isolation kit was the only extraction method that provided bands of the expected size and successful sequencing results. Although other protocols could generate high DNA quantities (Fig. 4), only DNA samples extracted with the PowerSoil kit could be amplified via PCR application (12 in a total of 40 samples). The success of PCR and sequencing did not depend on the used exoskeleton parts.

UV–Vis measurements to determine DNA concentration were performed as shown in Fig. 3. DNA concentrations were determined from the absorbance at 260 nm, which is the wavelength at which nucleic acids show an absorption maximum. Although samples extracted by ethanol precipitation methods showed a high amount of DNA according to UV–Vis measurements, such results were likely to be overestimated due to cross‐absorbance of humic acids at 260 nm. That type of contamination is commonly found in soil samples and usually coextracted with genomic DNA during the extraction procedure. Without proper separation techniques, the amount estimated by absorbance at 260 nm potentially included both genomic DNA and humic acids.

Failure in gene amplification from samples extracted by other protocols could be explained by low DNA yield left inside cicada exuviae after molt, and the inclusion of potential PCR‐inhibiting substances. Although the low DNA yield issue could be overcome by increasing the amount of template DNA, such a step might also enhance amplification of genomics of parasites such as fungi or bacteria, as shown in Fig. 2. We suspect that residual amounts of humic acids in DNA samples after extraction played a major role in preventing downstream applications. To circumvent this, the usage of PowerSoil DNA Isolation kit, which includes chemicals specialized in removing PCR inhibitors, is likely to reduce the residual amount of contaminants in DNA samples. Consequently, the A280/260 ratio of PS samples was found to be higher than those of other samples. Besides, due to the higher copy number of mitochondrial DNA than nuclear DNA, mt DNA might be more robust in gene amplification, even with the presence of PCR inhibitors. Hence, the decision on planned downstream analysis could affect the success of DNA extraction method.

We found that cicada exuviae are useful genetic materials in several respects. First, this material is easily collected as it is left on the surface after the individual casts its skin. Second, exuviae can be used as a reliable genetic source in studies where individuals are hard to catch and/or a high number of samples are required. Third, despite longtime exposure to variable environmental conditions, genomic DNA could still be extracted and was amenable to PCR applications. In this research, all exuviae were stored in room conditions for 14 months before DNA work, which possibly caused negative influences on quantity and quality of genomic DNA. Using fresh samples of within 48 hr after emergence, Lefort et al. (2012) report high success rate of 100%, whereas Kranzfelder, Ekrem, and Stur (2016) only receive highest target sequence success rate of 18%. Thus, we expect the usage of fresh exuviae or better preserved samples would enhance the amplification and sequencing successes. Furthermore, DNA from cicada exuviae could be applied for various genetic purposes, for instance species identification, investigation of genetic changes across consecutive broods, identification of unknown life cycles or determination of spatial‐ and temporal genetic diversities across populations. Subsequently, we expect our results will aid in genetic research of cicadas in the future.

## DATA ACCESSIBILITY

DNA sequences: Genbank accessions MF138135–MF138146.

## AUTHOR CONTRIBUTION

Hoa Quynh Nguyen contributed to research conception and design, conducted statistical analysis and data interpretation, and drafted the manuscript. Ye Inn Kim prepared data acquisition. Amaël Borzée contributed to research conception and design and provided critical revisions. Yikweon Jang provided critical revisions. All authors declare to have no conflicts of interests on this manuscript.

## CONFLICT OF INTEREST

None declared.
