# Corrigendum

**DOI:** 10.1002/ece3.3974

**Published:** 2018-03-26

Hoa Quynh Nguyen, Ye Inn Kim, Amaël Borzée, and Yikweon Jang (2017). Efficient isolation method for high‐quality genomic DNA from cicada exuviae. Ecology and Evolution. 2017; 7:8161–8169.

Please change the affiliations of

1. Hoa Quynh Nguyen

2. Ye Inn Kim

from: Department of Life Science, Ewha Womans University, Seoul, Korea

to: Interdisciplinary Program of EcoCreative, Ewha Womans University, Seoul, Korea

